# 'Bleeding Dilemma': The Story of a Periampullary Mass

**DOI:** 10.7759/cureus.3035

**Published:** 2018-07-23

**Authors:** Bhavyaa Bahl, Rohith Vadlamudi, Parekha Yedla, Roger D Smalligan

**Affiliations:** 1 UAB Internal Medicine, Huntsville Regional Medical Campus, Huntsville Hospital, Huntsville, USA; 2 UAB School of Medicine, Huntsville Regional Medical Campus, Huntsville Hospital, Huntsville, USA; 3 UAB Internal Medicine, Huntsville Regional Medical Campus, Huntsville Hospital, Huntsville , USA; 4 UAB Medicine, Huntsville Regional Medical Campus, Huntsville Hospital, Huntsville, USA

**Keywords:** cancer of ampulla of vater, periampullary mass, coil embolization, ampullary adenocarcinoma, gastroduodenal artery, gastrointestinal bleeding, angiography, melena, pancreatoduodenectomy, ampullectomy

## Abstract

Periampullary malignancies arise in the vicinity of the ampulla of Vater, a common passage for biliary and pancreatic secretions. Determining the anatomical origin of these tumors represents a diagnostic challenge. This is especially true for large tumors due to the transitional nature of this region, proximity to different structures, anatomical variations, and overlapping features among constituting structures. This determination has significant prognostic and therapeutic implications. Among them, primary ampullary adenocarcinoma is a rare malignancy that has the best overall prognosis with high rates of potentially curative resection and possible survival even in advanced disease. Due to its rarity, it is also a vague territory with no definitive guidelines regarding management and surveillance currently available. Acute gastrointestinal hemorrhage is a rare presentation of ampullary carcinoma that occurs secondary to tumor ulceration.

We report an elderly male with a previously known large, initially asymptomatic periampullary mass who came for evaluation of melena and was noted to be hypotensive secondary to acute blood loss from the large tumor, later determined to be adenocarcinoma of the ampulla of Vater.

## Introduction

The ampulla of Vater is an anatomically complex structure within the major duodenal papilla. It is a dilated passage at the confluence of the common bile duct and the main pancreatic duct, which drains their respective secretions into the second part of the duodenum through the papillary opening [1]. There are many anatomical variants of this arrangement. The sphincter of Oddi is a muscular valve consisting of smooth muscles surrounding the distal part of the common bile duct (CBD), the main pancreatic duct, and the ampulla. Tumors arising in this region are collectively referred to as periampullary and they may originate from any of the structures in the vicinity of the ampulla including pancreas, CBD, duodenum and the ampulla itself.

Despite being the most common site of neoplastic transformation in the small intestine, primary ampullary malignancies are rare with an incidence of four to six per million representing 0.5% of all gastrointestinal and 7% of all periampullary malignancies [1-3]. Some 90% of these are ampullary adenocarcinomas, which usually present as jaundice, pruritis, abdominal pain, nausea, dyspepsia, weight loss, melena, malabsorptive diarrhea, and fatigue. The strategic location of the tumor in the bile outflow tract leads to obstructive jaundice in up to 72%-90% cases allowing for the detection of small tumors early in the course of malignancy [4].

Both benign and malignant ampullary tumors can occur sporadically or in association with a genetic syndrome. The incidence of these tumors has shown dramatic increment in association with hereditary polyposis syndromes like familial adenosis polyposis (FAP) and hereditary non-polyposis colorectal cancer (HNPCC) [1]. These may be diagnosed earlier than the sixth or seventh decade of life, the average age of diagnosis for sporadic cases [1-2].

We present an elderly male with multiple comorbidities brought for evaluation of melena, a year after a prior incidence of hematemesis. Evaluation of hematemesis on prior admission had revealed a large periampullary mass that remained untreated. It was determined to be the cause of acute gastrointestinal hemorrhage leading to hemodynamic instability, a rare association with ampullary adenocarcinoma.

## Case presentation

A 79-year-old African American male was admitted for evaluation of two episodes of melena within one day. No associated abdominal pain, nausea, weight loss, appetite changes, diarrhea, hematemesis, or hematochezia was reported. His past medical history was significant for chronic obstructive pulmonary disease (COPD), heart failure with reduced ejection fraction of 25%, coronary artery disease, dementia, and a recent large left middle cerebral artery (MCA) stroke that had led to aphasia and residual right hemiparesis.

**Figure 1:**
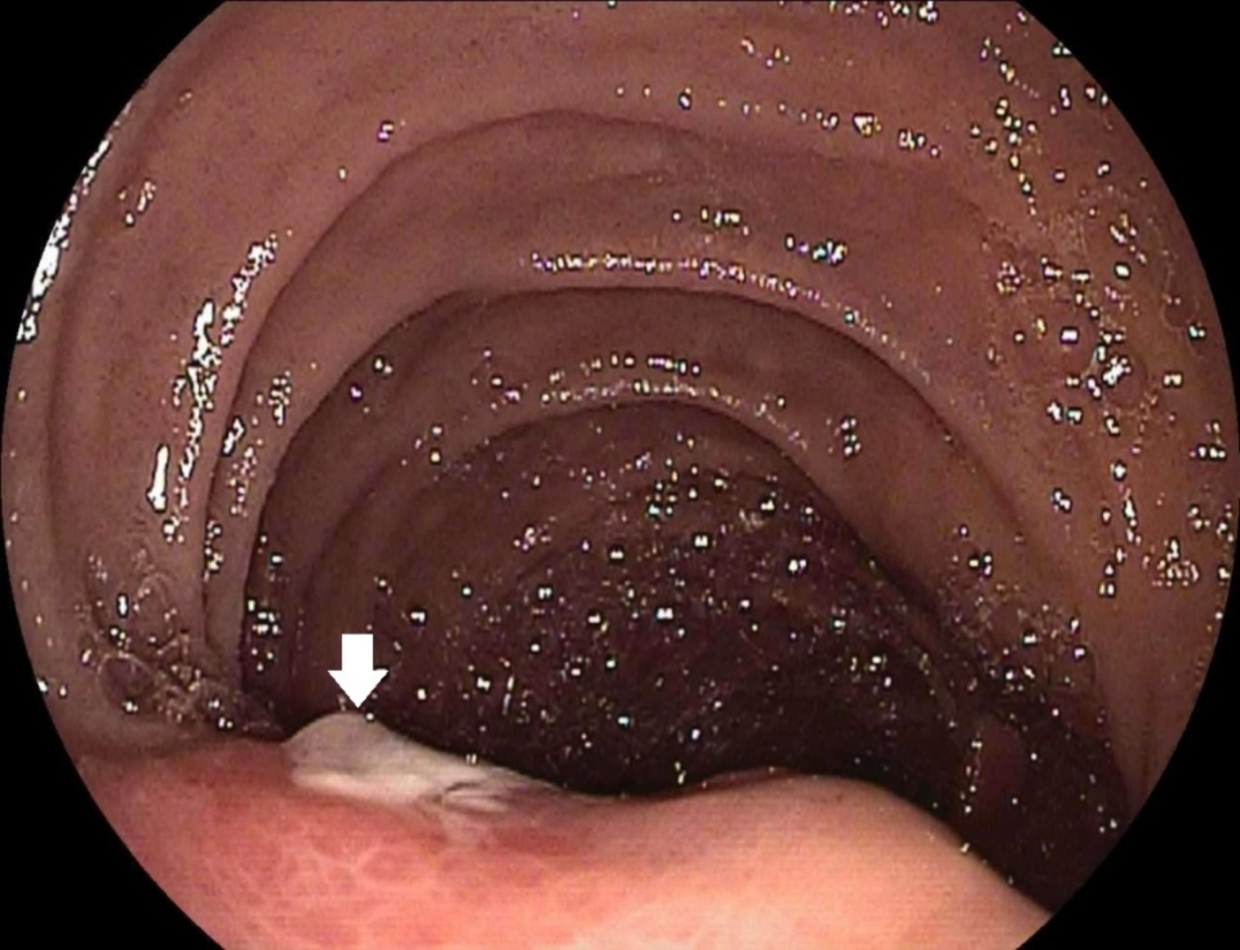
Esophagogastroduodenoscopy (EGD) on prior admission. Front-viewing endoscope showing an ulcerated submucosal mass at the duodenal papilla with no apparent bleeding.

The patient was admitted a year ago for evaluation of hematemesis with a hemoglobin level of 6.9 g/dL. At that time, esophagogastroduodenoscopy (EGD) had shown a large submucosal, ulcerated mass in the area of major duodenal papilla with histology suggestive of benign small intestinal mucosa without any atypical changes (Figure 1). A subsequent computed tomography (CT) scan of abdomen and pelvis confirmed a 6.7 cm x 5.5 cm mass at the pancreatic head invading the duodenum. It had led to a pancreatic duct dilatation of 11 mm seen as a cut-off sign on CT. Endoscopic ultrasound (EUS) to characterize the mass had to be terminated prematurely due to hypotension at the beginning of the procedure. He was eventually discharged after stabilization of his vitals and hemoglobin for a repeat outpatient EUS within a week. He failed to follow up with his appointment.

**Figure 2:**
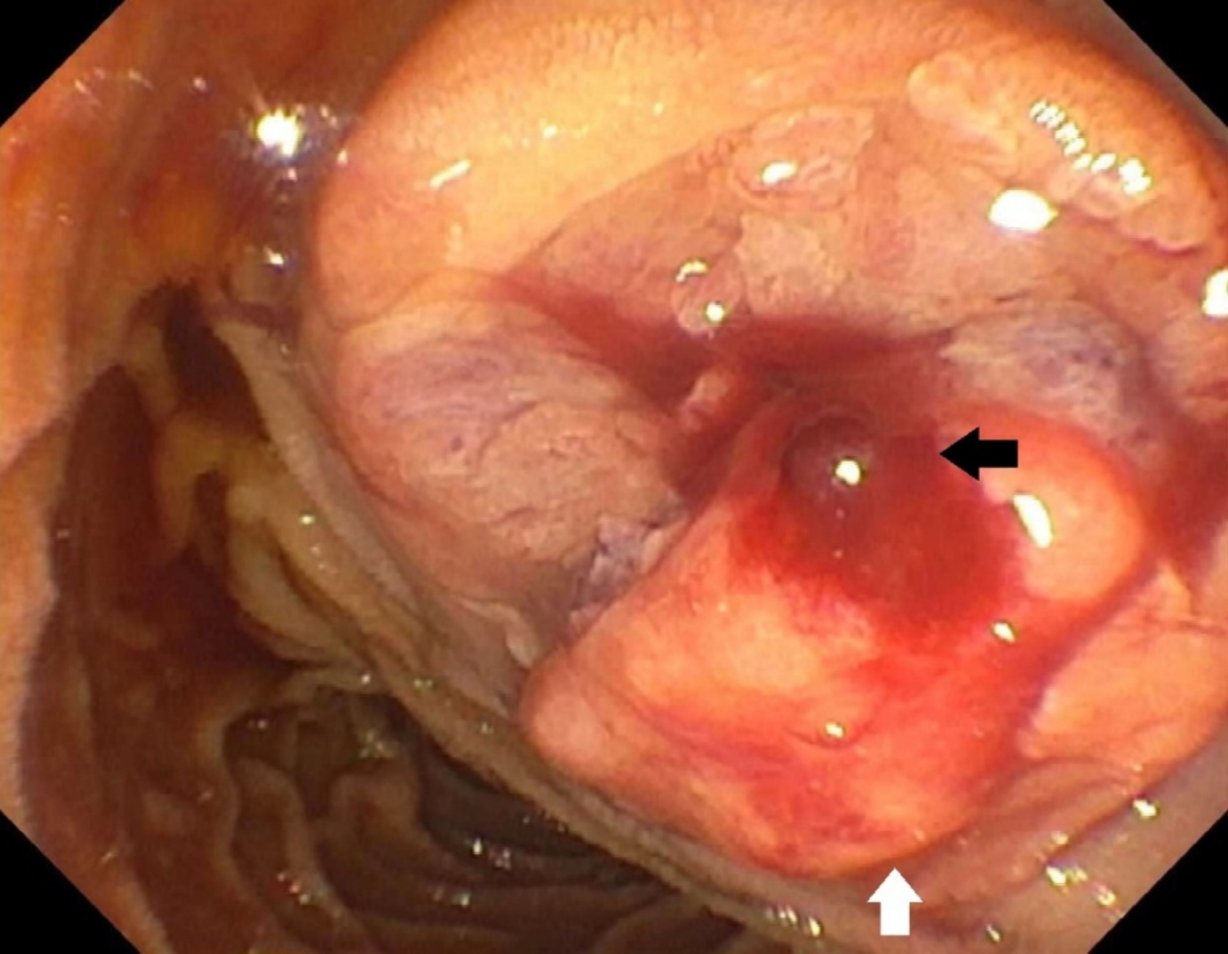
EGD on reported admission. Side-viewing endoscope showing a fungating, polypoid mass* (white arrow)* with blood oozing from the duodenal papilla *(black arrow)*.

Examination on this admission revealed an ill-appearing, aphasic, thin male with hypotension and tachycardia. Initial testing showed a hemoglobin level of 9.9 g/dL, a blood urea nitrogen (BUN) level of 30, an international normalized ratio (INR) of 1.1, and a total bilirubin level of 0.3. After initial resuscitation with intravenous fluids and red blood cell transfusions, an emergent EGD was performed using front- and side-viewing endoscope. A fungating, polypoid mass was seen within the ampulla of Vater with blood oozing out of the duodenal papilla that failed to be controlled with epinephrine injection (Figure 2). A hypervascular mass was seen within the second part of duodenum and pancreatic head with active hemorrhage from the supplying vessels including the superior pancreaticoduodenal branch of gastroduodenal artery (GDA) on the following arteriogram. Successful coil embolization of GDA was able to control bleeding and the patient did not require any further transfusions.

**Figure 3:**
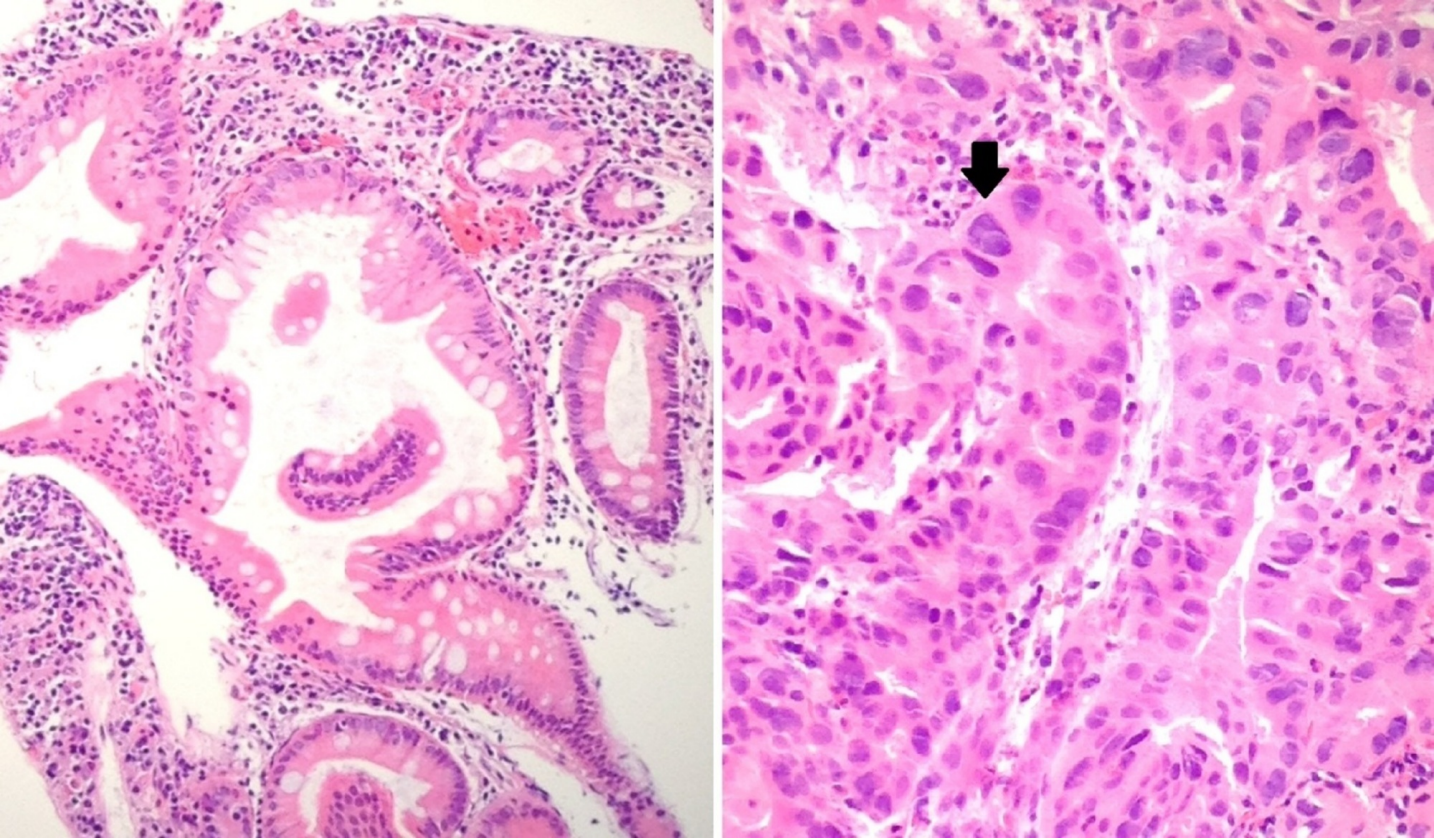
Histology of biopsy specimen from the ampulla of Vater using H&E, 100x. Left : Normal ampullary mucosa with intestinal differentiation. Right : Invasive ampullary adenocarcinoma with cellular atypia *(black arrow)*.

A biopsy specimen taken during endoscopy showed ‘invasive adenocarcinoma’ on histopathology (Figure 3). CA 19-9 level was within the reference range 15.6 U/mL (0-37 U/mL). Based on the clinical picture, imaging and pathology data, he was diagnosed with adenocarcinoma of the ampulla of Vater.

Due to his poor performance status and multiple comorbidities, he was not considered as a candidate for surgery or aggressive chemotherapy. He refused any aggressive measures and was discharged to a nursing facility with an outpatient oncology follow up for consideration of palliative radiotherapy.

## Discussion

Malignancies in the periampullary region are diagnosed based on the endoscopic, radiologic, and histopathologic data.

Ultrasound (US), CT, magnetic resonance imaging (MRI), magnetic resonance cholangiopancreatography (MRCP), or endoscopic retrograde cholangiopancreatography (ERCP) is used for evaluation of this region. The EUS is used only in a few cases requiring further delineation where there is suspicion for malignancy despite negative biopsy similar to the case described. It gives the most accurate assessment of the T stage along with tumor extension and penetration within the duct and surrounding tissues, respectively. The recent use of intraductal ultrasound (IDUS) to visualize and explore bile and pancreatic ducts has increased the diagnostic accuracy [5].

Large periampullary malignancies like the one presented, represent a diagnostic challenge in terms of determining the anatomical origin [6]. The importance of this determination has prognostic and therapeutic implications, which differ based on the tissue of origin. In such cases where the usual diagnostic modalities fail, a diagnosis may be reached once gross dissection and histopathology evaluation is performed after a complete surgical resection. Gross dissection to understand the relationship between the structures of these complex forms the basis of such a determination [6]. The clue to a particular origin may lie in the presence of a corresponding preinvasive disease on histological evaluation [6].

In this case, the tumor was considered to be ampullary and not pancreatic in origin due to a more indolent course of progression. Pancreatic adenocarcinomas have a highly aggressive course with a poor prognosis and a five-year survival rate of only three percent in contrast to 30%-50% noted in adenocarcinomas of ampullary origin with limited nodal involvement [1]. This malignancy was T3 tumor stage at the time of detection and did not show any progression a year later even in the absence of therapy.

Further histologic subdivision of ampullary adenocarcinoma into intestinal and pancreato-biliary subtype was first demonstrated by Kimura et al. in 1994 [7]. Histomolecular staining is now used to differentiate the two subtypes. This has been considered important due to a worse prognosis associated with the pancreaticobiliary subtype first demonstrated in a study by Westgaard et al. in 2008 although the evidence at this point is unclear [8-9]. The intestinal subtype stains positive for CK 20, MUC-2, and CDX-2 whereas the pancreaticobiliary subtype stains for CK 17, MUC-1 and MUC5AC, also shared by pancreatic adenocarcinoma (PDAC) [6]. In about five percent cases, a mixed pattern consisting of both intestinal and pancreaticobiliary glands is noted [6].

The 2017 American Joint Committee on Cancer (AJCC)/Union for International Cancer Control (UICC) TNM system is used for staging of ampullary adenocarcinoma. In nonmetastatic disease, prognosis mainly depends upon the extent of tumor invasion and lymphatic spread represented by the T and N stage, respectively.

Despite pancreatic duct obstruction secondary to a T3 stage tumor, our patient did not report any typical symptoms. These tumors are a rare cause of obscure gastrointestinal bleeding and more rarely, can also lead to overt hemorrhage secondary to tumor ulceration. This is seen as blood oozing out of the major duodenal papilla on a side-viewing endoscope which also facilitates visualization and biopsy of the ampullary mass. This may initially mimic other rare causes of obscure gastrointestinal bleeding (OGIB) including hemobilia and hemosuccus pancreaticus, which refer to bleeding originating in the CBD and pancreatic duct, respectively. The differentiation may become apparent during angiography when the source and tract of bleeding are delineated. The intermittent nature of bleeding in these conditions contributes to their obscurity. The CT angiogram (CTA), a noninvasive procedure has a sensitivity and specificity of 79%-90% and 95%-99% [10-11], respectively, in the detection of OGIB with a diagnostic accuracy of 100% for bleeding at a rate of 0.3-0.5 mL/min [12]. It is among the first-line diagnostic procedures that should be considered in a hemodynamically unstable obscure gastrointestinal bleed. Its major limitation is a lack of therapeutic application.

On the other hand, invasive angiography has both diagnostic and therapeutic applications. It can detect bleeding >0.5 mL/min with a sensitivity and specificity of 30%-47% and near 100%, respectively [13-14]. It may be used as an adjunct for therapeutic embolization after CTA in a hemodynamically unstable patient with brisk bleeding or as the only angiography procedure performed for both diagnostic and therapeutic purposes if the bleeding source is known and there has been a failure to achieve hemostasis despite the use of pharmacologic/endoscopic methods as described in the case discussed. Angiographic methods to control bleeding include injecting vasoactive agents like vasopressin or using agents to mechanically occlude the bleeding vessel, a process called embolization. Commonly used embolic agents are gelatin sponges, polyvinyl alcohol (PVA) particles, acrylic microspheres, and steel coils. Surgery is indicated in a few cases where the source is known, an increasing need for transfusions is noted, or there is life-threatening bleeding from a defined origin [12].

It is important to recognize the limitation of using a front-viewing endoscope while looking for the source of an OGIB as it can easily miss lesions of the ampullary complex due to its location. Endoscopic sampling is associated with a high false-negative histopathology nearing 50% due to sampling difficulties that could yield inadequate specimen. Side-viewing ERCP endoscope is able to identify causes in the gut wall and must be considered while evaluating the duodenal papilla and the ampullary complex.  

Pancreatoduodenectomy (PD) or Whipple’s procedure is the standard surgical procedure for ampullary cancer with potentially curative resection achieved in as high as 90% cases [15]. Five-year survival rates after PD with and without lymph node involvement are 17%-50% and 64%-80%, respectively [16]. Postoperative complications including pancreatic fistula, anastomotic leaks, delayed gastric emptying, intra-abdominal infections, and pneumonia are seen in up to 20%-40% cases [16]. The high postoperative morbidity limits the number of patients considered suitable to undergo PD.

Ampullectomy is an option that can be considered in early, limited lesions <1 cm when the patient is unsuitable to undergo PD, although it is not an alternative to it. An aggressive preoperative and intraoperative assessment is needed to rule out necessary criteria like tumor invasion, high-grade morphology, lymphovascular invasion, and lymph node involvement for such a consideration. The risk of tumor recurrence and shorter disease-free survival in comparison to PD is the most important limiting factor [17].

Ampullectomy can be performed as a minimally invasive transduodenal surgery or as an endoscopic procedure. Endoscopic ampullectomy is the surgery of choice for benign ampullary lesions <20 mm including lesions with low-grade dysplasia [18]. It may be considered in high-grade dysplasia and carcinoma in situ (Tis) after an appropriate staging and assessment. Lack of lymphadenectomy and proper pathological staging using this approach further increase the chances of tumor recurrence by many folds.  

Transduodenal surgical ampullectomy is an infrequently performed procedure which is less invasive than PD and has a lower surgical morbidity. It can be considered in Tis and T1 disease with lesions < 1 cm [17]. There are no uniform inclusion criteria for local resection. As a rule, PD should be performed whenever possible.

There is a lack of specific guidelines regarding the management and surveillance of this malignancy. Despite good resectability, recurrence remains a major issue. There is no consensus regarding the use of adjuvant chemotherapy due to the rarity of the condition, lack of definitive data, and uncertainty of benefits. However, most physicians offer adjuvant chemotherapy with (USA) or without (Europe) chemoradiation due to high recurrence rates. The drug regimen is on the lines of adjuvant chemotherapy for resected pancreatic adenocarcinoma based on fluoropyrimidine or gemcitabine [19-20]. Chemotherapy for metastatic disease based on the results of phase III ABC-02 trial consists of gemcitabine and cisplatin [19].

Poor prognostic factors include the presence of nodal metastasis, poor differentiation on histology, tumor invasion, and positive postoperative surgical margins. Other factors associated with an adverse outcome are macroscopic ulceration and perineural, lymphatic, or vascular invasion [15].

Despite a large number of studies regarding the management and prognosis of ampullary adenocarcinoma, there remains significant ambiguity evident by lack of treatment and surveillance guidelines. Our understanding remains limited due to the small sample size of these studies, rarity of the disease, and the dilemma posed by periampullary tumors. Hopefully, the increasing incidence as reported in a few studies will be met with defined guidelines in the near future [3].

## Conclusions

Tumors of the periampullary region represent a diagnostic challenge. Determining the tissue of origin in such cases is necessary for appropriate management and prognosis. Among these, ampullary cancer represents a rare category that has a more favorable prognosis than others. Acute gastrointestinal hemorrhage secondary to such a lesion is a rare presentation which may mimic conditions like hemobilia or hemosuccus pancreaticus requiring emergent management. Due to its rarity and lack of large multi-center trials, currently, there are no definitive guidelines for the management and surveillance of ampullary cancer. This has presented as an area of ambiguity for a disease considered to have a much better prognosis than all other periampullary tumors.
